# 

**Published:** 1896-01

**Authors:** 


### INDEX TO VOLUME L.

COMMUNICATIONS.

| | PAGE. |
|---|---|
| A Suggestion on the Origin of some Cases of Pyorrhœa Alveolaris, by Dr. H. C. Matlack | 230 |
| Address by the President, Dr. W. H. Todd, D.D.S. | 19 |
| Address by W. W. Shryock | 373 |
| Address at the opening of the Session of 1896–97, Dental Department of Howard University, | 573 |
| Address of Pres. J. Y. Crawford before the American Dental Association, | 582 |
| A Labor of Love | 396 |
| An Anomalous Condition | 508 |
| A National Dental Library and Museum, by Wm. Donnally | 43 |
| Antiseptics, by Dr. Geo. E. Hunt | 365 |
| A Rare Case, by H. L. Ambler, M.D., D.D.S. | 39 |
| A Suggestion, by L. P. Bethel, M.D., D.D.S | 234 |
| A Porcelain Inlay Ground to Fit a Cavity in a Tooth, by A. W. Harlan, M.D., D.D.S | 277 |
| A Suggestion about Cocaine, by Geo. E. Hunt, D.D.S. | 40 |
| Benefit of Dental Society Meetings, by Dr. D. E. Delzell | 404 |
| Care of Children's Teeth, | 600 |
| Chairman's Address, by M. H. Fletcher, M.D., D.D.S. | 105 |
| Common Ground of Dentistry and Medicine, by Joseph Roach, M.D | 161 |
| Dental Education, by W. T. Jackson, D.D.S. | 29 |
| Dental Examining Boards in the United States, and Preliminary Education, | 59 |
| Dentistry a Distinct and Independent Profession, by R. Finley Hunt, D.D.S. | 116 |
| Dental Vulcanite, by W. Storer How, D.D.S | 268 |
| Diagnosis of Lesions of the Heart Previous to Administering Anæsthetics, by W. H. Whitslar, D.D.S. | 22 |
| Discussion of a Paper on "The Dentist in his Profession among the People," by Dr. S. B. Hartman | 200 |
| Dental Caries During Pregnancy, by H. F. Vandervort, D.D.S | 280 |
| Ethics, by S. B. Brown, D.D.S. | 209 |
| Electro-Therapeutics, | 590 |
| Four Requisites in Development, by M. F. Ault, M.D., D.D.S. | 13, 53 |
| Introductory to Discussion of Dental Nomenclature, by Dr. S. B. Brown | 507 |
| Lining Root-Canals, by L. P. Bethel, M.D., D.D.S. | 417 |
| Materia Medica and Therapeutics, by J. S. Cassidy, M.D., D.D.S. | 91, 422 |
| Michigan State Board of Examiners in Dentistry | 282 |
| Necrosis, by Dr. N. W. Hiatt. | 375 |
| New Method for Pyorrhœa Alveolaris | 165 |
| Physicians Should Do Less Work | 508 |
| Physiological Antagonisms, by Dr. Geo. E. Johnson | 389 |
| Physiological Antagonisms between Medicine and Disease | 113 |
| Population of Greater New York | 276 |
| Progressive Calcification, by Junius E. Cravens, D.D.S. | 224 |
| Report of Committee on Dental Science and Literature | 505 |
| Some Thoughts on the Combination of Amalgam and Gold, by Dr. F. W. Knowlton | 26 |

PAGE.

Shadowgraphs without the Crooke's Tubes, by John L. Gish, M.D., D.D.S...... 121

Then and Now, by J. A. Robinson, D.D.S.................. 396

The Dentist, by Dr. B. F. Shepard........................ 406

The Disinfection of Pulpless Teeth, ......................... 584

The Electrical Aspect of Cataphoresis, by L. E. Custer, B.S., D.D.S.............. 261

The Value of Differential Diagnosis in Dentistry, by V. A. Latham, M.D........ 313

The Absorption of the Roots of the Temporary Teeth, by Allison Wm. Haidle, D.D.S......................... 388

The Bridge Case—Decision of the Court...................... 237

The X-Rays in Dentistry, by F. W. Sage, D.D.S......... 272

The Pathology of Inflammation, by Geo. E. Hunt, D.D.S......................... 1

The Use of Compressed Air in Dentistry, by S. Freeman. 34

The Necessity of, and Methods for Better Pulp Protection in Filling Teeth, by J. D. Patterson, D.D.S..... 41

The Offices and Eccentricities of the Dental Pulp, by D. D. Smith...................... 44

The Originator of the Quinine Industry...................... 120

The Destruction of Children's Teeth, Cause and Prevention, by J. G. Henister, M.D.......................... 157

The Literary Side of our Profession, by Dr. Wm. H. Steele ......................... 216

The Good and the Objectionable Qualities of Oxyphosphate of Zinc as a Filling Material, by C. A. Quackenbush ............... 220

What Should the Dentist Do to Prevent Decay of the Teeth? by N. S. Hoff, D.D.S.......................... 379

PAGE.

What Difficulties Does the Dentist Encounter from the Presence of Secondary Formations? by E. F. Day, D.D.S.................. 222

PROCEEDINGS AND MEETINGS.

Fifty-Third Annual Meeting of the Mississippi Valley Association of Dental Surgeons ..... ................ 501

National Association of Dental Faculties.................. 449

Proceedings of Meeting of the Michigan State Dental Association...............469, 592

Points of Interest in the Case of Children's Teeth, by B. J. De Vries, D.D.S......... 521

Personal Observations of the Development of Dentistry and Dental Legislation in Michigan, by G. E. Corbin, M.D., D.D.S............ 548

The American Medical Association ........................ 427

The Mississippi Dental Association ......................... 322

Southern Dental Association ........... ....................67, 363

MONTHLY DIGEST.

Reported by Mrs. J. M. Walker.......328, 487, 568, 609

SELECTIONS.

A Blow at the Germ Theory... 290

A Case of Unusual Speech Defect ......................... 294

Aconite to Abort Colds ......... 180

Aluminum in the United States......................... 296

Alcohol and Happiness......... 131

Alcohol as a Panacea............ 199

Alcohol in the Treatment of Disease ....................... 511

A Long-Lived Family........... 169

A New Dressing in Surgery... 296

A New Picture Process.......... 177

A Needed Reform................. 515

Anything to Get Well........... 134

PAGE.

A Report of the Committee on the Abuse of Alcoholic Drinks from a Sanitary Standpoint.................... 200

Arsenical Poisoning............. 140

Asbestos as a Surgical Dressing.............................. 459

A Superfluous Detail........... 247

A Slight Misunderstanding.... 178

Asbestos ............................ 181

A Slap at the Bacteriologists.. 183

Baby's Rights ..................... 290

Biting the Nails................... 138

Capacity of Women for Bearing Pain ......................... 460

Care of the Hair.................. 297

Camphor for Ether Collapse... 246

Child Study......................... 127

Cheerfulness........................ 184

Children's Ailments and Household Preventatives, 47

Chlorate of Potash as a Tooth-Powder ........................ 190

Chloroform Anæsthesia Dangerous to Meat-Eaters...... 299

Classification of Diseases...... 169

Composition on Physiology.... 298

Comparative Vitality of the Sexes............................ 133

Decline of Alcohol as a Medicine, by T. D. Crothers, M.D................................ 197

Deodorization of Iodoform..... 172

Digestible food ................... 459

Early Rising and Insanity..... 150

Effectual Treatment of Acute Alcoholism.................... 129

Elimination through the Stomach ........................ 141

Electrical Effects of Sprays..... 187

Face Reading....................... 192

Fate of a Famous Health Resort................................ 136

Fatigue .............................. 516

Fooled by his Imagination..... 504

French Physicians on the Drink Problem.............. 615

Good Breeding...................... 299

Growth in the Nasal Passages Due to Cold .................. 514

Guaiacol as a Local Anæsthetic ............................ 99

PAGE.

Hardening Steel by Gas ........ 195

Heredity ............................ 6

Heroic Treatment for Drunkards ............................ 293

Higher Medical Education..... 294

How Austraia Deals with Drunkards ..................... 297

How to Avoid Colds.............. 101

How Carborundum is Made... 285

How to Sterilize Cotton......... 295

Hygiene of the Face.............. 240

Ill-Temper a Symptom of Excessive Meat Eating........ 136

Infective Inflammation of the Parotid Glands............... 170

Insomnia. ......................189, 296

Is the Bacteriological Theory of Disease Waning?........ 182

Kerosine in Surgery....... ...... 457

Lime Water ......................... 180

Liquid Air.......................96, 142

Manners of Doctors............... 98

Medical College Association... 292

Medical Students in Paris...... 190

Medicine as a Moral Agent.... 515

Milk from Sewage Farms...... 512

Moving Toward the Light...... 526

Municipal Housekeeping in Paris................................ 102

Neuralgia........................... 142

New Method of Administering Chloroform..................... 97

Notes on Tuberculosis—Avenues of Invasion, by Jas. T. Whittaker, M. D. ........ 614

Odor as a Symptom of Disease 358

Ohio Tobacco Law ... ........... 292

Onions................................ 299

Overdone Sterilization. ........ 100

Over-supply and Diminished Demand ........................ 100

Painless Tooth Extraction...... 181

Poisonous Action of the Aqueous Vapor of Expired Air 102

Possibilities of the X-Rays in Surgery ........................ 185

Potassium Iodide.................. 133

Pregnancy and Dental Caries.. 138

Quantity of Food.................. 461

Resorption of the Salts of Iron 247

Role of the Nerves in the Mind Cure............................ 288

PAGE.

Second Pan-American Medical Congress.......................... 292

Separation of Teeth...........95, 143

Shun Bolted Flour, Why Americans Lose their Teeth at an Early Age, by John Ellis, M.D........................ 124

Solutions............................ 130

Specific Treatment of Necrosis of the Alveoli and Maxillæ with Aromatic Sulphuric Acid, by W. A. Mills, D.D.S........................... 173

Surgery without Pain........93, 147

Surgical Uses of Cocaine...132, 513

"Ter Die"........................... 178

The Best Antiseptic............... 139

The House We Live In......... 99

The Hygienic Importance of Amusement........................ 291

The Influence of the Mind...... 179

The Influence of Odors Upon the Voice........................ 192

The Nerves of Taste............ 463

The Official Language at the International Med. Congress at Moscow in 1897... 292

The Preventive of Consumption............................. 137

The Retro-buccal Method of Exposing the third Branch of the Trigeminus........... 293

The Risks of Anæsthesia........ 140

The St. Petersburg School of Medicine for Women...... 181

The Sanitation of Workshops and Public Conveyances, by B. Merrill Ricketts, M.D 241

The Secret of Centenarianism.. 145

The Turkish Bath................ 167

The Treatment of Burns........ 188

The Triumphs of Electricity... 144

The Use of Vinegar to Prevent Vomiting after Chloroform Inhalation............ 179

Treatment of Alcoholism by Strychnine Nitrate......... 295

The Use and Abuse of the Brain........................94, 149

Tobacco Insomnia..................... 46

To Open the Eustachian Tube 103

To Estimate the Dampness of a House........................ 144

PAGE.

Unprofessional Conduct......... 139

Washington City Dental Society.............................. 150

What Becomes of the Microbes? 196

What is a Poison?................ 194

What is Man's Natural Food? by C. E. Page, M.D......... 509

What is the Possible Minimum Death Rate?................ 291

### COLLEGE COMMENCEMENTS

American College of Dental Surgery.............................. 346

Atlanta Dental College......... 350

Baltimore College of Dental Surgery........................... 303

Birmingham Dental College... 306

Boston Dental College........... 355

Chicago College of Dental Surgery.............................. 345

Cincinnati Academy of Dentistry.......................302, 413

Cincinnati College of Dental Surgery........................... 350

Columbian University, Dental Department.................... 349

Chicago Northwestern University, Dental Department.. 301

Cleveland University of Dentistry and Surgery, Dental Department..................... 304

Detroit College of Medicine.... 410

Harvard Dental College......... 352

Indiana Dental College......... 303

Kansas City Dental College. . 309

Louisville College of Dentistry 356

Meharry Medical College, Dental Department............... 308

Missouri Dental College......... 300

National University, Dental Department .................. 356

New York College of Dentistry 351

Ohio College of Dental Surgery.............................. 349

Ohio Medical University, Dental Department ............. 345

Pennsylvania College of Dental Surgery.................... 307

Philadelphia Dental College... 304

Richmond University, College of Medicine.................... 411

PAGE.

Royal College of Dental Surgeons of Ontario..... ....... 348

The American Academy of Dental Science............... 311

The Odontological Society...... 311

Toronto Royal College of Dental Surgeons.................. 412

University of Buffalo, Dental Department......... ........ 302

University of California, College of Dentistry............ 353

University of Denver, Dental Department.................. 355

University of Iowa, Dental Department.................... 308

University of Maryland, Department of Dental Surgery................ .......... 301

University of Michigan, College of Dental Surgery... 353

University of Pennsylvania, Department of Dentistry 357

University of Tennessee, Dental Department.............. 352

University College of Medicine, Dental Department.. 354

Vanderbilt University, Dental Department........... ...... 306

Western Dental College of Kansas City.................. 347

Western Reserve University, Dental Department....357, 412

### NOTICES.

American Dental Association .............................360, 361

American Medical Association, Section of Dental and Oral Surgery............ 248

National Association of Dental Faculties................. 360

Notice..........................248, 249

Pacific Coast Dental Congress. 48

Twelfth International Medical Congress ................ 361

### EDITORIAL.

Alabama State Dental Association ............... ............ 205

PAGE.

American Dental Association.. 464

American Medical Association, Dental Section...... 206

Another Dental College....... ... 519

Another Mistake.................. 520

Annual Meeting of the Indiana State Dental Society........ 362

Bibliographical...207, 257, 259, ..........................518, 570

Biographical........................ 413

Book Notice.........................363

Change of Place................... 309

Dr. Jack's Views on Implantation............................ 572

Educational College Work..... 517

Inter-State Dental Meeting..... 310

Mississippi Valley Dental Association...................156, 254

National Dental Library and Museum. ...................... 251

National Association of Dental Faculties................. 465

Ohio State Dental Society...51, ............................467, 571

Ohio State Dental Law......... 152

Popular Education in Dentistry............ .............. 255

Editorial.............................. 103

Taking the Air Without Going Out............................ 416

The Dental Colleges ............ 48

The Dental Register............. 49

The Editorial "We"........... 206

The Medical Law................ 152

The National Association of Dental Faculties............ 569

### OBITUARY.

Dr. Harvey Scott................. 153

W. H. Dwinelle..................... 154

S. R. Bingham....................... 155

Dr. R. H. Wilson.................. 208

C. W. Spalding,........... ........ 364

### IN MEMORIAM.

Dr. W. H. Dwinelle..............

Dr. P. G. C. Hunt, ...........257, 413

Dr. G. H. Kinney, .............. 259

### DIRECTORY OF DENTAL SOCIETIES.

*American Dental Association*—Meets first Tuesday in August, 1896. At Saratoga, N.Y. Pres., J. Y. Crawford, Nashville, Tenn.; Rec. Sec., Geo. H. Cushing, Chicago.

*Southern Dental Association*—Next meeting will be held at Atlanta, Ga., November 5th, 1895. Pres., H. E. Beach, Clarksville, Tenn. Sec., S. W. Foster, Decatur, Ala.

### STATE DENTAL SOCIETIES.

*Alabama Dental Association*—Meets annually. Next meeting at Selma, on the second Tuesday of April, 1896. Pres. A. A. Pearson, Montgomery; Sec. S. W. Foster, Decatur.

*California State Dental Association*—Next meeting will be held in San Francisco, second Tuesday in June, 1895. Pres., L. A. Teague, San Francisco; Rec. Sec., W. Z. King, San Francisco; Cor. Sec., C. E. Post, San Francisco.

*Colorado State Dental Society*—Meets 1st Tuesday in June, 1894, at Glenwood Springs. Pres. P. T. Smith, Denver; Sec., Sarah May Townsend, Denver.

*Connecticut State Dental Society*—Next meeting will be held in Hartford, third Tuesday in May, 1895. Pres. Chas. P. Graham, Middletown; Rec. Sec. Geo. L. Parmele, Hartford.

*Dental Society of the State of Maryland and District of Columbia*—Meets annually. Next meeting at Washington City, on the second Tuesday in October, 1893. Pres. B. F. Coy, of Baltimore: Sec. M. W. Foster, Baltimore.

*Florida State Dental Association*—Meets annually. Next meeting at Tampa, 1st Tuesday in May, 1895. Pres, W. A. McQuaig, Lake City; Cor. Sec. S. W. Allen, Tampa.

*Georgia State Dental Society*—Meets at Indian Spring, in 1895. Exact time to be decided later; Pres. W. W. Hill, Washington; Rec. Sec. S. H. McKee, Americus; Cor. Sec. O. H. McDonald, Atlanta.

*Illinois State Dental Society*—The next meeting will be held at Springfield, Ill., May 12 to 15, 1896. Pres. W. A. Stevens, Chicago., Sec. Louis Ottofy, Chicago.

*Indiana State Dental Society*—Will meet at Detroit, Mich., on the third Tuesday of June, 1895. Pres. D. S. Harrold, Elwood; Secretary, G. E. Hunt, Indianapolis.

*Iowa State Dental Society*—Meets annually. Next meeting at Iowa City, May, 7th to 10th, inclusive, 1895. Pres. J. S. Kulp, Muscatine; Rec. Sec. F. T. Breene, Iowa City.

*Kentucky State Dental Association*—Meets annually. Next meeting in Louisville, Ky., 3rd Tuesday in June, 1896. Pres., J. H. Letcher, Danville; Sec., J. H. Baldwin, Louisville.

*Kansas State Dental Association*—Pres. C. B. Reed, Topeka; Sec., W. W. West, Topeka. Next meeting will be held at Topeka, second Monday in May, 1895.

*Maine Dental Society*—Will meet on the third Tuesday in July, 1895, in Bangor. Pres. T E. Tibbetts, Rockland; Sec. F. A. Knowlton, Fairfield.

*Maryland State Dental Association*—Next annual meeting will be held (place not definitely known yet,) in April, 1895. Pres., Chas. C. Harris, Baltimore; Sec, W. W. Dunbracco, Baltimore.

*Massachusetts Dental Society*—Meets annually in Boston, in June. Pres. Josiah W. Ball, Boston; Rec. Sec., E. O. Kinsman, Cambridge.

*Michigan State Dental Association*—Meets annually. Next meeting at Detroit, on the 3rd., Tuesday of June, 1896. Pres., J. Ward House, Grand Rapids; Sec. ————. Treas. Geo. H. Mosher, Jackson.

*Mississippi Dental Association*—Next meeting date to be fixed, 1896, at Jackson, Miss. Pres. T. C. West, Natchez; Sec. L. N. Jeffries, Natchez.

*Minnesota State Society*—Pres. E. B. Weeks, Leitchfield; Sec., H. L. Cruttenden, St. Paul. Will meet in Winona, in September, 1896.

*Missouri Dental Association*—Meets at Bertle Springs, Mo., second Tuesday in July, 1895. Pres., J. T. Fry, Moberly; Sec., W. W. Carter, Sedalia.

*Nebraska State Dental Society.*—Next annual meeting will be held at Norfolk, third Tuesday in May, 1895. Pres., H. W. Shriver, Omaha; Cor. Sec., O. M. Huestis, Nebraska City.

*New Jersey State Dental Society*—Meets annually. The next meeting will be held August, 1896. Pres. R. M. Sanger, E. Orange; Sec. C. A. Meeker, Newark.

*New Hampshire Dental Society*—Meets Annually. Next meeting will be held in Concord, N. H.; time to be decided later. Pres. W. R. Blackstone, Manchester; Sec. Burton C. Russell, Keene.

*North Carolina State Dental Society*—Meets in Salisbury, on the second Tuesday in May, 1895. Pres. H. H. Harper, Kinston; Sec., J. E. Wyche, Greensboro.

*North Dakota State Dental Society*—The next meeting will be held at Fargo, May, 1895. Pres. E. M. Pierce, Hillsboro; Sec. C. L. Rose, Fargo.

*Ohio State Dental Society*—Meets in Columbus, first Tuesday in December, 1896. Pres. Hervey Barnes, Cleveland; Sec. L. P. Bethel, Kent.

*Pennsylvania State Dental Society*—Will hold its next annual meeting at Eagle's Mere, Pennsylvania, July 9th, 1895. Pres., Dr. J. A. Libbey, Pittsburgh; Cor. Sec., H. E. Roberts, Philadelphia.

*Rhode Island Dental Association*—Pres., C. M. Noble, Providence; Sec., H. W. Gillett, Newport, Meets quarterly, second Tuesday in July, October, January and April. Annual Meeting second Tuesday in July, at Newport.

*South Carolina State Dental Association*—Meets annually. Pres. J. C. Orland, Spartanburg; Cor. Sec. C. B. Colson, Charleston. Next meeting at Spartanburg, October, 1895.

*Tennessee Dental Association*—Meets annually. Next meeting at Knoxville, July 9, 1895. Pres. W. H. Richards, Knoxville; Rec. Sec. W. P. Houston, Lawrenceburg; Cor. Sec. W. J. Morrison, Nashville.

*The Dental Society of the State of New York*—Meets annually on the second Wednesday in May. Next session at Albany, 8th and 9th of May, 1895. Pres. F. T. Van Woert, Brooklyn; Sec. C. S. Butler, Buffalo; Cor. Sec. R. Ottolengui, New York.

*Texas State Dental Association*—Meets in San Antonia, third Tuesday in May, 1894. Pres. A. A. Beville, Waco; Sec. Chas. B. Lewis, Ennis

*Vermont State Dental Society*—Meets annually. Next meeting will be held at Brandon, Vt., third Wednesday in March, 1895. Pres. W. H. Wright, Brandon; Sec. Thos. Mound, Rutland.

*Virginia State Dental Association*—Will meet in 1895. Exact time and place to be decided later. Pres. H. W. Campbell, Suffolk; Sec. J. Hall Moore, Richmond.

*Washington State Dental Society*—Meets Annually. Next meeting will be held at Seattle, in the middle of May, 1895. Pres. B. S. Scott, Ellenburg; Sec., R. B. Gentle, Olympia.

*Wisconsin State Dental Society*—Meets annually. Next meeting will be held at Madison, Wis., the third Tuesday in July, 1895. Pres., Chas. C. Chittenden, Madison; Sec., C. A. Southwell, Milwaukee.

*West Virginia State Society.*—The annual meeting will be held at Parkersburg, on the first Wednesday of October, 1894. Pres. J. N. Mahan, Charleston; Sec. Geo. I. Keener, Morgantown.

### LOCAL SOCIETIES.

*American Academy of Dental Science*—Meets annually in Boston, Mass., on the first Wednesday in October. Pres. J. H. Batchelder, Rec. Sec. William Y. Allen.

*Atktnsonian Dental Society of Chicago*—Meets second Tuesday each month. Pres. J. G. Reed; Sec. C. E. Meehoff.

*Buffalo Dental Association*—Meets last Monday evening of each month. Annual meeting in June. Pres. F. H. Boswell; Sec. J. A. Stackhouse.

*Brooklyn Dental Society and Second District Society United*—Meets quarterly—Pres. T. H. Holly; Rec. Sec. A. N. Russell, Brooklyn.

*Central Dental Association of Northern New Jersey*—Meets bi-monthly; and annually. The next annual meeting will be held about the middle of February, 1895. Pres. Thomas Moore, Paterson; Sec. Wm. L. Fish, Newark.

*Chicago Dental Society*—Meets monthly. Pres. W. V. B. Ames; Sec. A. H. Peck;

*Chicago Dental Club*—Meets on the fourth Monday of each month. Pres. I. D. Sperling; Sec. E. L. Clifford.

*Cleveland Dental Society.*—Meets the first Monday of each month. Pres. J. R. Bell; Sec. J. F. Stephan.

*Connecticut Valley Dental Society*—Meets annually. Next meeting in Oct. 1895, time and place to be decided later. Pres. C. C. Barker, Meriden, Conn.; Sec. Geo. A. Maxfield, Holyoke, Mass.

*Detroit Dental Society*—Meets monthly, Pres. Geo. L. Field, Sec. A. W. Diack.

*Dental Society of Southwestern Michigan*—Meets at St. Joseph, October 8th and 9th, 1895. Pres. S. M. White; Sec. E. I. Backers.

*Eighth District Dental Society, N. Y.*—Next annual meeting April 24th, 1894, in Buffalo. Pres. F. J. Burkhart, Batavia; Sec., W. V. Grove, Buffalo.

*East Tennessee Dental Association*—Meets annually. Next meeting will be held at Harriman, second Tuesday in June 1895. Pres. F. A. Shotwell, Rogersville; Sec. and Treas. Geo. C. Brause, Harriman.

*Eastern Illinois Dental Society*—Meets annually.

*Fifth District Dental Society of N. Y.*—Meets semi-annually. Next annual meeting will be held in Utica, 2d Tuesday in April, 1895. Pres. Frank Jones, Syracuse; Cor. Sec. A. R. Cooke, Syracuse.

*First District Dental Society of the State of New York*—Meets annually. The next meeting will be held on the second Tuesday of April 1895. Pres., Victor Hugo Jackson, New York City; Sec., Benj. C. Nash, New York City.

*First District Dental Society of Illinois*—Next meeting will be held in Canton, second Tuesday in September, 1895. Pres. W. A. Johnston, Peoria; Sec. W. O. Butter, La Harpe.

*Hayden Dental Society of Chicago*—Meets third Monday each month. Pres. T. E. Powell; Sec. A. J. Oakey.

*Knoxville City Dental Society*—Meets monthly. President, J. T. Cazier; Sec. A. J Cottrell.

*Minnesota State Dental Society*—Annual Meeting will be held in St. Paul, 2nd Wednesday in Sept., 1895. Pres. C. H. Robinsin, Wabasha; Sec. W. L. Cruttenden, Northfield.

*Mississippi Valley Dental Society*—Meets annually at Cincinnati. Next meeting March, 1896. Pres., W. V. B. Ames, Chicago; Rec. Sec. H. T. Smith, Cincinnati; Cor. Sec. H. C. Matlack, Cincinnati, O.

*New England Dental Society*—Meets annually. Next meeting 2nd Thursday in Nov., 1895, in Boston Mass. Pres. J. F. Adams, Worcester, N. H.; Sec. E. O. Kinsman, Cambridge, Mass.

*Northern Illinois Dental Society*—Meets annually. Next meeting at Aurora, October 17th, 1895. Pres. T. W. Beckwith, Sterling; Sec. J. W. Cormany, Mt. Carroll.

*Northern Indiana Dental Society*—Meets annually third Tuesday in October. Pres. E. J. Church; Sec. C. G Keekn.

*Odontological Society of Pennsylvania*—Meets on the first Saturday evening of each month, at 8 o'clock p. m., at 13th and Arch sts; Pres. Jas. Truman, Rec. Sec. A W. Deane.

*Odontological Society of New York City*—Meets the third Tuesday of each month. Pres. Dr. A. L. Northrop; Cor. Sec. Dr. George A. Wilson.

*Odontological Society of Cincinnati*—Meets monthly. Pres. G. Molyneaux; Sec. H. C. Matlack.

*Odontological Society of Chicago*—Meets third Tuesday each month. Pres. S. A. Swasey; Sce. and Treas., Louis Ottofy.

*Odontographic Society of Chicago*—Meets second Monday each month. Pres. Dr. M. Gallie; Sec. H. H. Wilson.

*Pennsylvania Association of Dental Surgeons*—Meets second Tuesday of each month. Pres. H. E. Roberts; Cor. Sec. Theo. F. Chupein.

*Pittsburg Dental Society*—Meets the last Tuesday Evening of each month. Pres. J. B. Williams, Homestead; Sec. N. L. Reinecke.

*Quincy, (Ills.) Dental Society*—Meets Monthly.

*San Francisco Dental Society*—Meets the second Monday evening of each month. Annual Meeting in October. Pres. C. E. Post; Sec. G. N. Van Orden; Cor. Sec. H. G. Richards.

*Sixth District Dental Society of N. Y.*—Meets semi-annually. The annual meeting will be held at Binghamton, the first week in May, 1895. Pres., M. H. Fish, New Berlin; Sec., F. W. McCall, Binghamton.

*Seventh District Dental Society of Ohio*—Meets annually. Next meeting in May, 1894. Pres., O. N. Heise, Cincinnati; Sec., J. R. Callahan, Cincinnati.

*Southern Illinois Dental Society*—Meets annually. Next annual meeting will be held on the 3d Tuesday in October, 1896. Pres., Sec., G. W. Entsminger, Carbondale.

*Southern Minnesota Dental Society*—Next meeting in Mankato, third Tuesday in April, 1895. Pres. S. Bond, Onoka: Sec. F. B. Kremer, Minneapolis.

*Stomatological Club of California*—Meets in Sanfrancisco. Pres. W. J. Younger.

*St. Louis Dental Society*—Meets first and third Tuesday in each month. Pres. J. B. Newby; Cor. Sec. John G Harper: Rec. Sec. F. F. Fletcher.

*The Isaac Knapp Dental Coterie of Fort Wayne, Ind.*—Meets the first Thursday of each month. Pres., W. W. Mungen; Sec., M. A. Mason.

*The Minneapolis Dental Society Monthly*—Pres. E. F. Clark, Sec. E. J. Morrison.

*The Tacoma Dental Society*—Meets monthly. Pres. M.C. Burns; Sec. W.E. Burkhart,

*Washington City Dental Society*—Meets 3d Tuesday of each month. Pres. Dr. J. Roland Walton; Sec. Dr. D. Elmer Wiber.

### The Dental Register.

With the present number The Dental Register enters upon its fiftieth volume, the closing year of its half century. What it has accomplished during this time for the profession, need not here be specified, this, with any good degree of accuracy, could not be done.

The *American Dental Journal* preceded it eight years; during this interval, viz: from 1839, the establishment of the American Dental Journal to 1847, the establishment of the Dental Register, there were seven dental journals established, two in London, and five in the United States. All of these with two or three exceptions had a very brief existence. The New York *Dental Recorder* continued for ten years, from 1846 to 1856, when it ceased. Stockton's *Dental Intelligencer* was published two years. The *Forceps*, of London, England, was published two years, the other four but one year each.

Thus it will be seen that the *American Dental Journal* and the DENTAL REGISTER are the only journals now in existence, having their original names. The *Dental News Letter* was established in the same year as the DENTAL REGISTER, but after twelve years it was changed to the *Dental Cosmos*.

The American Journal was suspended from 1860 to 1867, so that the REGISTER has continued without break or intermission longer than any other dental journal in existence, having never failed in a single issue.

The late Dr. James Taylor, of Cincinnati, was the editor for the first nine years, then for fifteen years it was edited by J. Taft and George Watt; since the twenty-fifth volume it has been conducted by the present editor; up to 1859 it was issued as a quarterly, since that time as a monthly.

The aim of the REGISTER has been and is, to give matter of permanent value to the profession, and to make record so far as practicable of the progress that is being made in the various lines of developmental work.

The pages of the REGISTER are open to all contributions that are of interest and value to the profession.

---

At the annual meeting of the Odontographic Society of Chicago, held December 9, 1895, the following officers were elected for the ensuing year:

President, Dr. C. E. Meerhoff; Vice-President, Dr. E. R. Carpenter; Secretary, Dr. H. H. Wilson; Treasurer, Dr. Edmund Noyes.

Board of Directors: Dr. R. B. Tuller, Dr. C. E. Bentley, Dr. J. G. Reid.

Board of Censors: Dr. A. B. Allen, Dr. H. A. Drake, Dr. G. W. Schwartz.

### CONTENTS.

COMMUNICATIONS.

The Pathology of Inflammation ........ 1

Four Requisites in Development ........ 13

Address by the President, Dr. W. H. Todd, D.D.S. ........ 19

Diagnosis of Lesions of the Heart Previous to Administering Anæsthetics ........ 22

Some Thoughts on the Combination of Amalgam and Gold ........ 26

Dental Education ........ 29

The Use of Compressed Air in Dentistry ........ 34

A Rare Case ........ 39

A Suggestion about Cocain ........ 40

The Necessity of and Methods for Better Pulp Protection in Filling Teeth ........ 41

A National Dental Library and Museum ........ 43

The Office and Eccentricities of the Dental Pulp ........ 44

SELECTIONS.

A Safeguard ........ 45

Tobacco Insomnia ........ 46

Children's Ailments and Household Preventitives ........ 47

Local Anæsthesia ........ 47

NOTICES.

Pacific Coast Dental Congress ........ 48

EDITORIAL.

The Dental Colleges ........ 48

The Dental Register ........ 49

Officers Elected at Odontographic Society ........ 50

Ohio State Dental Society ........ 51

Obituary ........ 52

### THE DENTAL REGISTER,

**A MONTHLY MAGAZINE DEVOTED TO THE INTERESTS OF THE**

### DENTAL PROFESSION.

*Terms Reduced to $2.00 Per Year. Single Copies, 25 Cents.*

**EDITED BY PROF. J. TAFT, D.D.S.**

—AND—

**Published by SAM'L A. CROCKER & CO., Ohio Dental and Surgical Depot.**

The volume commences in January and closes in December.

The REGISTER being issued monthly is always fresh, therefore Indispensable to the practitioner who desires to keep posted up with the new inventions and improvements which are daily developed. Neither expense nor effort will be spared to give value and variety to its contents. Articles will be illustrated with well executed engravings whenever they will add to the interest or assist to explanation.

Its extensive circulation and frequency of issue make it one of the BEST DENTAL ADVERTISING MEDIUMS in the United States.

All subscriptions must begin with January or July.

Local or special notices, five lines or less, will be inserted for $3 each insertion; over five lines, 50 cts. per line.

All advertising bills payable in advance, or at furthest, after the issue of the first number containing the advertisement. All transient advertisements to be paid for in advance.

☞CONTRIBUTIONS SOLICITED.

Exclusively Editorial Communications should be addressed to the Editor.

The latest number of the REGISTER may be ordered with or without other goods any time, and all letters should be addressed to

